# Supplementary material for: Microscopic phage adsorption assay: High-throughput quantification of virus particle attachment to host bacterial cells
Source: Proc Natl Acad Sci U S A. 2024 Dec 19;121(52):e2410905121. doi: 10.1073/pnas.2410905121 (PMC11670125; doi:10.1073/pnas.2410905121)
Supplement: Supplementary file 1 — Appendix 01 (PDF) [file pnas.2410905121.sapp.pdf]

PNAS

Supporting Information for

Microscopic Phage Adsorption Assay: High-throughput quantification of virus particle attachment to host bacterial cells

Jyot D. Antani*, Timothy Ward, Thierry Emonet, Paul E. Turner*

* Corresponding authors: Jyot D. Antani, Paul E. Turner

Email: jyot.antani@yale.edu; paul.turner@yale.edu

This PDF file includes:

- Tables S1 and S2
- Supplementary Notes
- Figures S1 to S6
- Legend for Movie S1
- Extended Materials and Methods
- SI References

Other supporting materials for this manuscript include the following:

- Movie S1

Supplementary Tables

Table S1. Morphology and microscopic outcomes for the phages used in this study.

**Phage**	**Host**	**Capsid Diameter (nm)**	**Particle Length (nm)**	**Fluorescent foci visibility**
T4	*E. coli*	120	80	✓
λ	*E. coli*	60	150	✓
T5	*E. coli*	90	160	✓
M13	*E. coli*	7	880	✓
T2	*E. coli*	114	115	✓
T7	*E. coli*	60	20	✓
ϕX174	*E. coli*	25	25	×
P1	*E. coli*	85	228	✓
A1-1	*S. flexneri*	97	143	✓
OMKO1	*P. aeruginosa*	139	210	✓

Table S2. Bacterial strains used in this study. (*Eschericia coli K-12* unless otherwise specified).
* CGSC# refers to the catalog number at the Coli Genetic Stock Center at Yale University, now available through *E. coli* Genetic Resource Center (https://ecgrc.net/).

Phage	Strain abbreviation	Strain number*
T4	WT^{T4}	Keio collection Δ*icdC*, CGSC #11220
	Δ*ompC*	Keio collection, CGSC #9781
	Δ*waaC*	Keio collection, CGSC #11805
	Δ*waaF*	Keio collection, CGSC #10648
	Δ*waaO*	Keio collection, CGSC #10652
	Δ*waaR*	Keio collection, CGSC #10651
	Δ*waaG*	Keio collection, CGSC #10655
T2	WT	Keio parent strain BW25113
	Δ*ompF*	Keio collection, CGSC #8925
	Δ*fadL*	Keio collection, CGSC #9875
	Δ*fadLompF*	JM1101, CGSC #5844
T5	WT	BW25113
	Δ*fhuA*	Keio collection, CGSC #8416
T7	WT	BW25113
	NS1	CGSC #6517
	NS2	CGSC #6518
M13	F-pilus	CSH22
	ΔF-pilus	BW25113
P1	Ca^{2+}	BW25113
	No Ca^{2+}	BW25113
λ	WT	BW25113
	Δ*lamB*	Keio collection, CGSC #10877
A1-1	M90T	*Shigella flexneri* M90T
	M90T Δ*ompA*	*Shigella flexneri* M90T Δ*ompA*
OMKO1	*P. aeruginosa*	*Pseudomonas aeruginosa* PAO1
	E. coli WT	BW25113

Supplementary Notes

Supplementary Note 1. Whole Genome Sequencing of Keio strains

**Strain**	**deleted genes***
WT^{T4}	*icdC*
Δ*ompC*	*ompC*
Δ*waaC*	*[waaF],waaC,[waaL]*
Δ*waaF*	*[waaG],waaQ*
Δ*waaG*	*[waaG],[waaQ]*
Δ*waaO*	*waaR,[waaB]*
Δ*waaR*	*[waaY],waaJ,[waaR]*

* Square brackets represent partial deletions and no brackets represent full deletions.

The sequence of LPS-related genes as in the MG1655 gemone on biocyc.org is shown below:

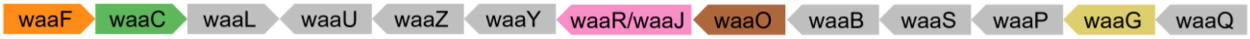

In the whole genome sequence of our ancestral strain BW25113 annotated with GenBank accession number CP009273, we observed the following sequence:

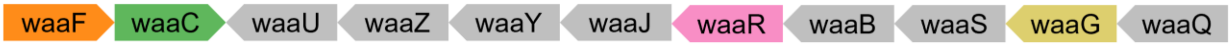

Mutations in each of our test strains were identified via the breseq mutant calling algorithm (1), and are listed in the table above. The differences in the waaJ-waaR-waaO region may explain the discrepancies in the final two rows of the above table: waaO and waaR are likely annotated as waaR and waaJ respectively.

Supplementary Note 2. Area under survival curve represents mean trajectory duration

For individual trajectory duration T, survival probability as a function of time t:

$$F(t) = P(T > t)$$

then,

$$f(t) = -F'(t)$$

where, $f(t)$ is the probability density function, such that

$$\int_0^\infty f(t)\,dt = 1$$

Area under survival curve,

$$AUC = \int_0^\infty P(\tau > t)\,dt = \int_0^\infty F(t)dt$$

$$= \int_0^\infty F(t) \cdot 1\,dt$$

$$= t \cdot F(t)\,\Big|_0^\infty - \int_0^\infty t \cdot F'(t)\,dt$$

$$= 0 + \int_0^\infty t \cdot \big(-F'(t)\big)\,dt$$

$$= \int_0^\infty t\,f(t)\,dt$$

$$= \langle T \rangle$$

Supplementary Note 3. Commentary on the assumption that adsorption reaction is pseudo first-order

A common model for phage adsorption assumes that the free phage depletion rate depends on the concentration of both bacteria B and phages P , with adsorption rate constant k (2–4):

$$\frac{dP}{dt} = kBP \qquad \text{Equation 2}$$

Solving this equation with initial condition $P(t = 0) = P_0$ yields

$$k = -\frac{1}{Bt}\ln\left(\frac{P}{P_0}\right) \qquad \text{Equation 3}$$

A number of studies in literature report adsorption rate constant k with units [mL min^{-1}], obtained through the above model. B is assumed to be constant throughout the 10-minute experiment because B is in large excess of P. It is assumed that a 1.4-fold difference in B (assuming fast growth, 20-minute doubling time, which results in 1.4-fold change in concentration within 10 minutes) does not have a huge impact on the bacterial surfaces that the phages have available for attachment (5). However, if B is included in the calculation of k, the measurement errors in B contribute to the errors in k (**Fig S6**).

Following the same argument, under our experimental condition where B (~0.5-5×10^8 CFU/mL) is in large excess relative to P (~1-5×10^4 PFU/mL), an assumption of pseudo first-order phage depletion is fair (6), where B is assumed to be constant in the model, and hence kB in Equation 2 is replaced by an effective adsorption rate constant k_{eff} [min^{-1}], which yields

$$\frac{dP}{dt} = k_{eff}P \qquad \text{Equation 4}$$

This assumption has been experimentally and argumentatively validated by classic literature (5, 7) and discussed by recent literature on phage adsorption (4). Solving Equation 4, we get Equation 1 (main text):

$$P = P_0 \exp\left(-k_{eff}\, t\right)$$

We followed this assumption in order to get a less error-prone estimate of rate constant from the classic adsorption assay (see replicate distributions and error-bars in **Fig S6**A versus those in **Fig 3**C), which we could compare with the microscopy assay outcomes.

Supplementary Figures

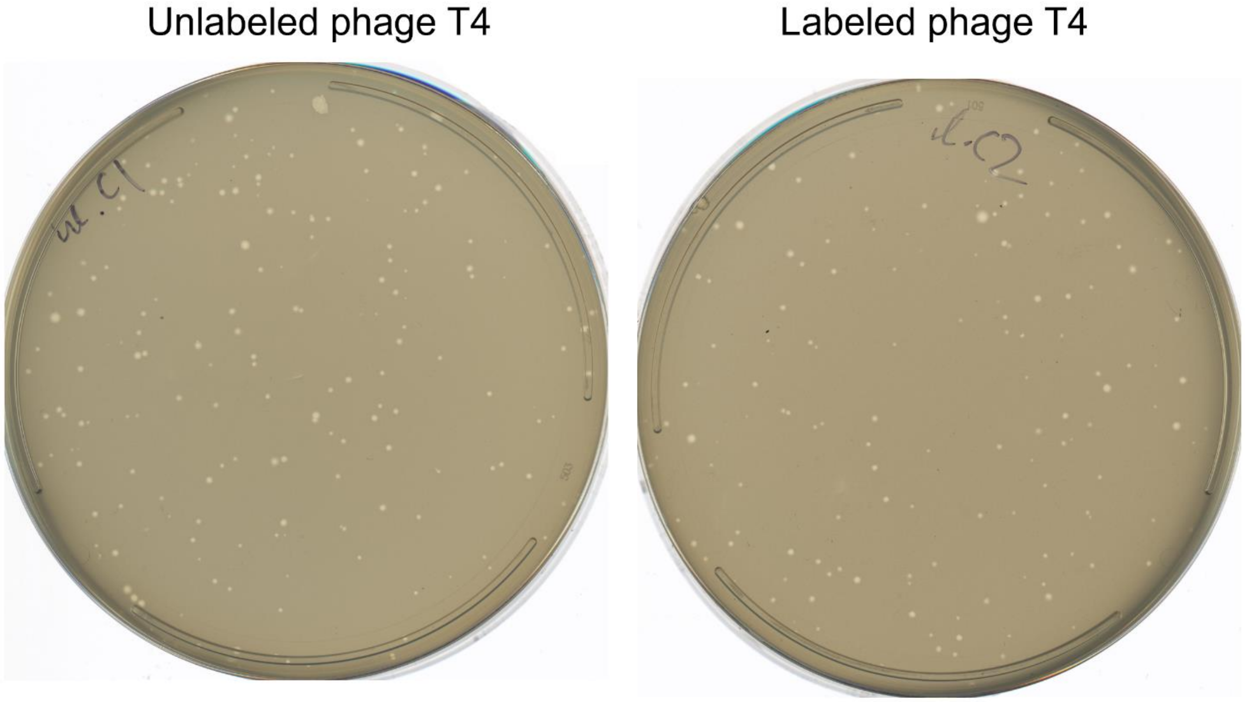

Fig S1. Labeled phages retain their infectivity. Plaque assays were performed with labeled and unlabeled phage T4 with host BW25113. Labeled phages yielded plaques on lawns of the bacterial hosts, indicating successful infections. We also performed similar experiments with all other phages used in this study (phages λ, T5, M13, T2, T7, P1, A1-1, and OMKO1) and observed identical results (data not shown).

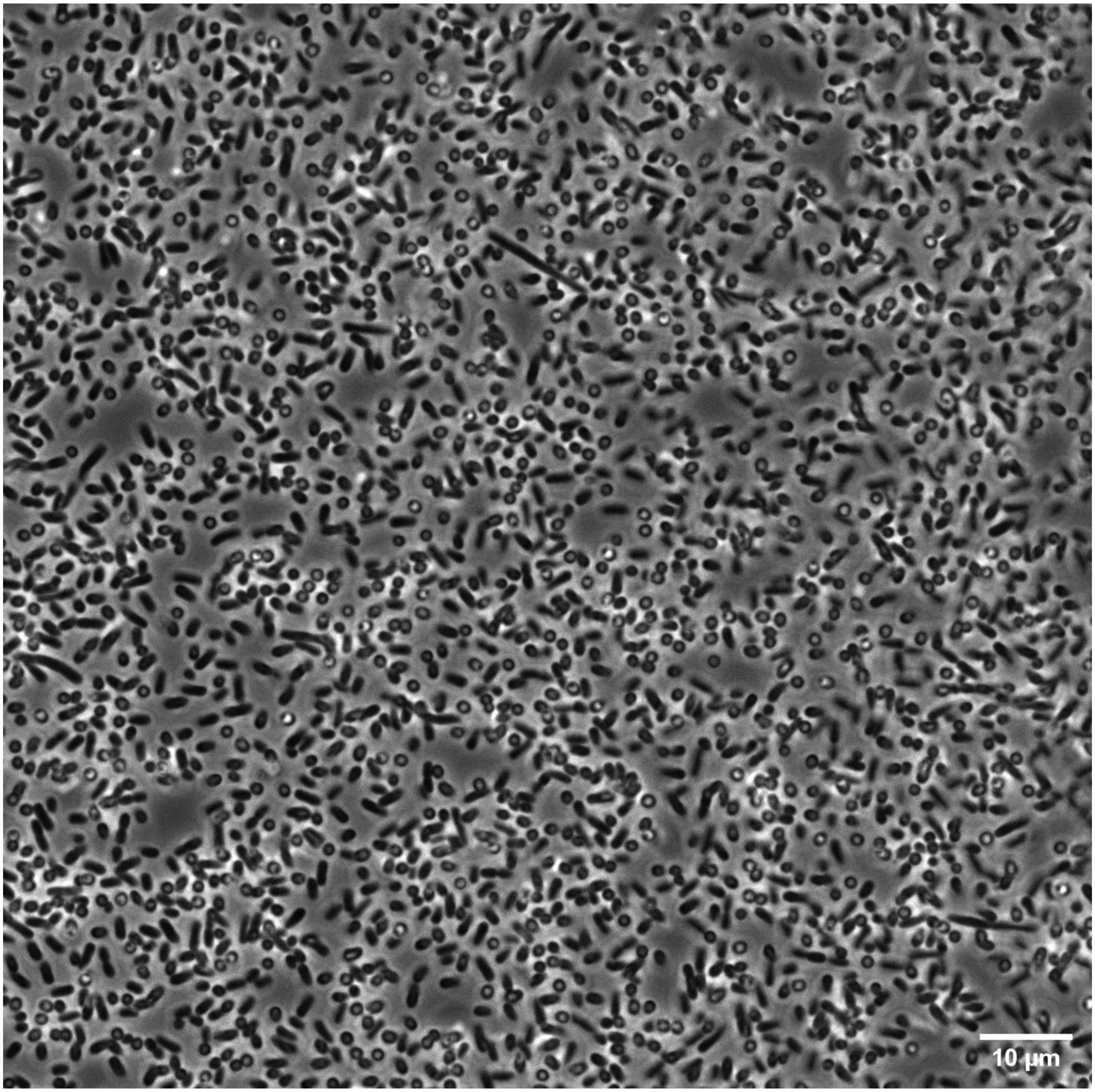

Fig S2. Lawn of closely packed bacteria immobilized on the coverslip. Phages were introduced to this lawn after immobilizing bacteria on the glass coverslip with 0.01% poly-L-lysine.

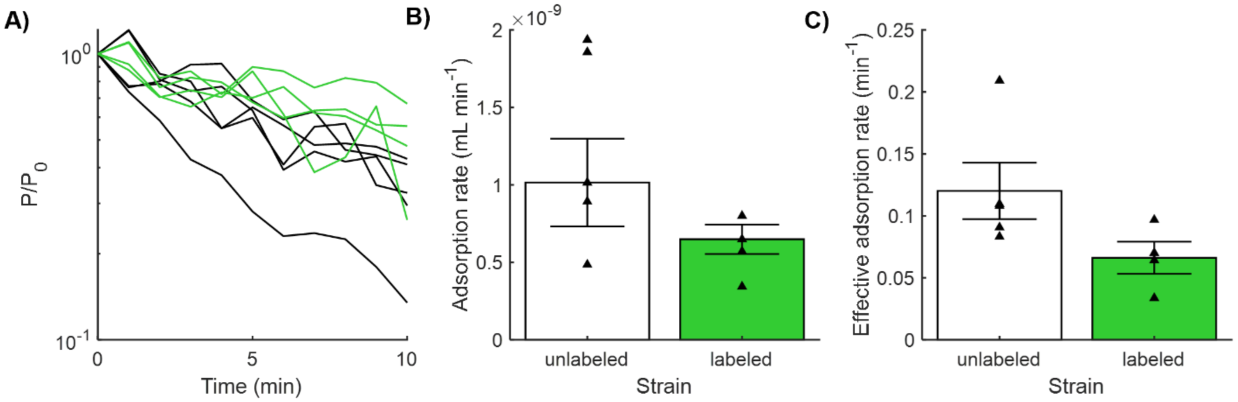

Fig S3. Classical adsorption assay revealed that adsorption rates of labeled and unlabeled phages are comparable. (A) Classic adsorption curves for T4 phages, unlabeled (black lines; n = 5 replicates) or labeled with the lysine-specific dye (green lines; n = 4 replicates) are indicated. Estimates of the adsorption rate constant $\boldsymbol{k}$ **(B)** and the effective adsorption rate constant $\boldsymbol{k_{eff}}$ **(C)** showed no statistically significant differences, when comparing labeled and unlabeled phages. Statistical significance was assessed by Student's t-test, which yielded P = 0.09 in each comparison. Plots depict means ± standard errors.

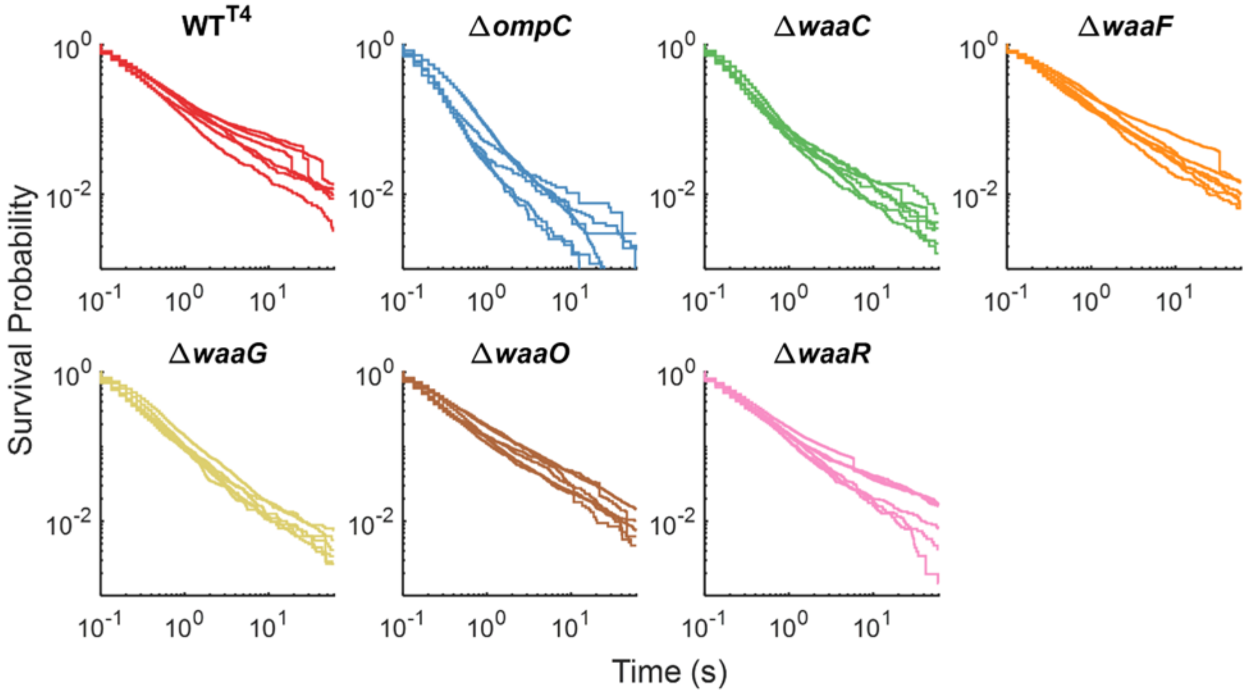

Fig S4. Survival probability curves (n = 6 replicates) for phage T4 on each bacterial strain shown in **Fig 2**D.

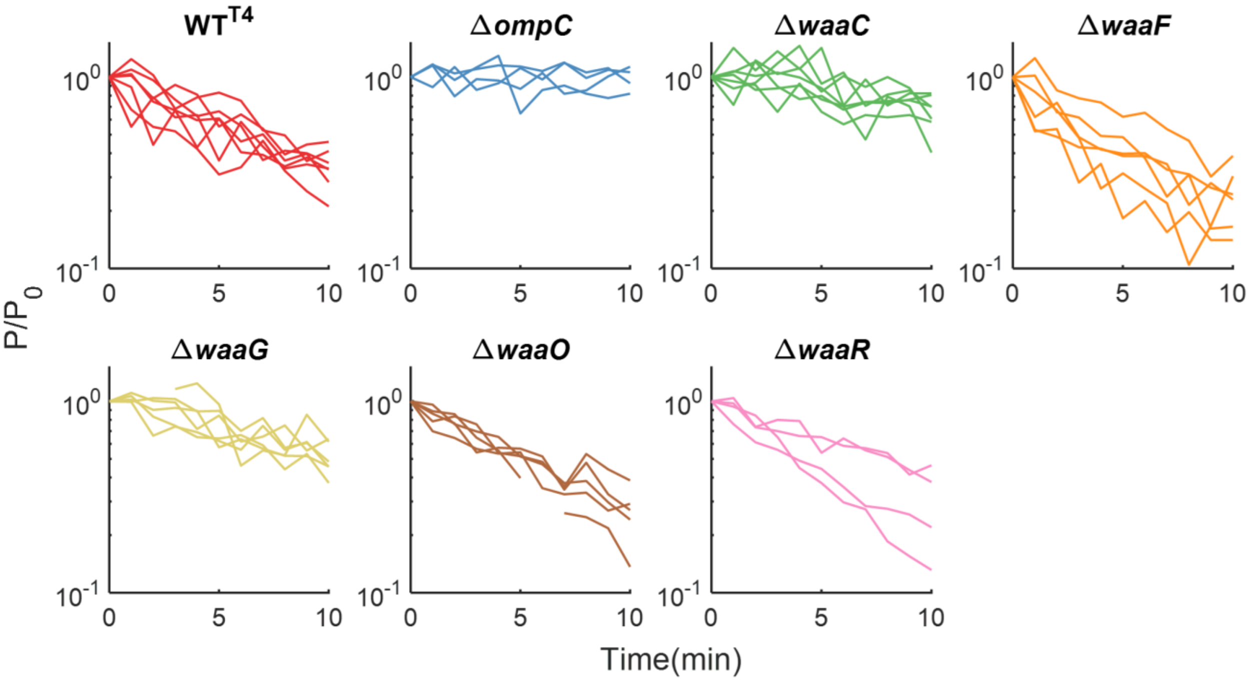

Fig S5. Classic adsorption curves (n = 4 to 6 replicates) for phage T4 on different host strains of *E. coli* shown in **Fig 3**C.

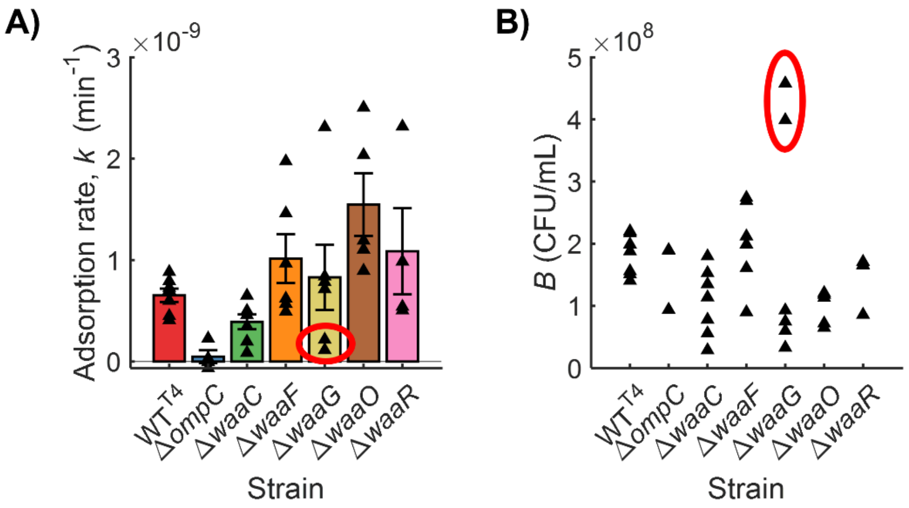

Fig S6. Adsorption rate constant k has a higher measurement variability due to measurement variability in bacterial concentration. (A) Adsorption rate constant k was calculated for each experimental replicate as $k = -\frac{1}{Bt}\ln\left(\frac{P}{P_0}\right)$, which accounts for the bacterial concentration B. **(B)** Bacterial concentration measured for each experimental replicate is plotted. The datapoints within the red circles exemplify how extreme values of B in the experiment influence the calculated value of k.

Movie S1 (separate file). After addition of fluorescently labeled phages, a majority of bacteria in the microscopic focal view visibly undergo lysis.

Extended Materials and Methods

Bacteria and phage strains

All *E. coli* K-12 bacteria and phages of *E. coli* used in this study (**Table S1**, **Table S2**) were kindly provided by J. Wertz at the Coli Genetic Stock Center (CGSC) at Yale University, which is now *E. coli* Genetic Resource Center (https://ecgrc.net/). Strain BW25113 was used as the wildtype (WT) *E. coli* host for phages T2, T5, T7, P1 and λ, whereas derivatives of BW25113 in the Keio collection were mutants that contained individual gene knockouts and were used as challenge hosts due to genetic changes in cell-surface receptors (**Table S2**). For phage T4, a mutant with knockout in pseudogene *icdC* was used as a proxy for wildtype (WT^{T4}). For phages M13, A1-1, and OMKO1, strains *E. coli* CSH22, *S. flexneri* M90T, and *P. aeruginosa* PAO1, respectively, were used as wildtype hosts; either a knockout of these hosts or strain BW25113 (WT) was used as the challenge host lacking the cellular receptor for each of these viruses (**Table S2**). Phages A1-1 and OMKO1 have been described previously (8, 9).

Lysogeny broth (LB; 10 g tryptone, 5 g yeast extract, 10 g NaCl per L) was used for liquid cultures, 1.5% agar plates, and 0.75% top agar for the double-layer method of phage propagation. With exception of experiments using phage T4 and phage P1 interacting with WT in absence of Ca^{2+}, LB was supplemented with 25 μg/mL thymine, 10 mM $MgSO_4$, and 5 mM $CaCl_2$. 0.1% D-(+)-Maltose was further supplemented to the growth and experimental media for phage λ. Overnight cultures were initiated from colonies grown on solid (1.5%) LB agar and placed into LB liquid medium, incubated with shaking (175–200 RPM) at 37°C. Serial culture was conducted by diluting 0.1 mL overnight culture into 10 mL fresh LB medium. Densities of bacteria were determined by plating dilutions from cultures onto agar plates, to estimate colony-forming units (CFU) per mL. Bacterial stocks were stored in 25% glycerol at -80°C. Phages were enumerated (plaque-forming units [PFU] per mL) using the standard double-layer agar method where viruses form visible plaques on confluent lawns of wildtype host bacteria within 0.75% top agar, overlayed on 1.5% agar in the bottom layer. High-titer stocks (lysates) of phages were grown using the double-layer agar method: top agar layers with a lacey plaque-pattern were suspended in PBS, centrifuged, and filtered (0.22 μm) to remove bacteria and obtain a cell-free lysate.

Phage labeling with fluorescent dye

Fluorescent labeling of phage T4 with Alexa Fluor® 488 NHS Ester dye was previously described (10). An improved dye-amine conjugate, TFP (tetrafluorophenyl) ester, a more hydrolysis-resistant amine-reactive ester as compared to NHS (succinimidyl) ester, was utilized in the current study: AZDye™ 488 TFP Ester from Fluroprobes (Catalog No. FP-1026, Alexa Fluor® 488 TFP Ester equivalent) was used in all labeling protocols. While dye wavelength (488 nm) was chosen for compatibility with our in-house fluorescence microscopy filter sets, we have successfully used the same labeling protocol for dyes excitable at other wavelengths or offering conjugation-chemistry other than amine-reactive esters. Amicon® Ultra-4 centrifugation filter columns with a cutoff size 100 kDa were used to transfer ~10^8-10^9 phages from a lysate to phosphate buffer saline (PBS, pH 7.4, Gibco™). The 100 kDa cutoff was sufficient to filter out components in the media while retaining phages. Transferring phages to PBS ensured robust dye-conjugation as competing growth-media components carrying lysine molecules were removed before introducing the dye. The phages were then conjugated with 0.5 mg/mL dye via shaking (500 rpm) incubation at room temperature for 3 hours, followed by static incubation at 4 °C overnight. Excess unbound dye was removed via centrifugation (4-5 washes) using another Amicon® Ultra-4 filter. Labeled phages were stored at 4 °C until imaging. Phages were imaged within three weeks of conjugation in order to avoid lower fluorescence signal due to dye degradation.

Microscopy (MPA assay)

Single phages were visualized via epifluorescence microscopy at high spatiotemporal resolution. A Nikon Ti-E inverted microscope equipped with a 1.40 NA objective with 100× magnification,

perfect focusing system, and temperature-controlled chamber was used. The temperature was maintained at 34 °C throughout the experiments. Tunnel slides (glass slides with coverslips affixed at edges via double-sided adhesive tape) were used to prepare samples. In the experiments measuring free phage diffusion, the objective was focused ~10 μm above the coverslip surface. For live phage-bacteria interaction assays (the MPA assay), 0.01% poly-L-lysine was first introduced to the tunnel slide, which was then incubated upside down for at least 5 and up to 30 minutes, to coat the coverslip. Exponential-phase bacteria from freshly-grown cultures were washed twice in PBS, concentrated, and flowed into the tunnel slide, followed by a 10-minute upside down incubation to adhere and immobilize the bacteria. After washing away unadhered bacteria with the growth medium, fluorescently-labeled phages suspended in LB growth medium (as it contains attachment cofactors such as tryptophan (11, 12)) with the appropriate supplements described above, were introduced to the tunnel slide. The tunnel-openings were immediately sealed with VALAP (equal parts Vaseline, Lanolin, and Paraffin Wax) to avoid evaporation and flows, and the sample was mounted onto the microscope for imaging. The objective was focused ~0.5 μm above the coverslip surface, identified via visualization of phages immobilized at (attached to) the poly-L-lysine-coated surface. A snapshot of underlying bacterial lawn was recorded in the phase-contrast channel. Finally, videos of phages attaching to bacteria were recorded in the fluorescence channel at a high temporal resolution of 30 frames per second. To ensure comparability between experiments, experimental replicates of the same phage interacting with different host/non-host strains were obtained on the same day.

Analysis of microscopy videos to obtain single phage trajectories

From videos of fluorescently labeled phages recorded at 30 frames/s, trajectories of single phage particles interacting with host cells were obtained using custom-written MATLAB algorithms for particle tracking, similar to algorithms described earlier (13–15). Our algorithm is described below, which is equivalent to popular particle tracking programs such as `trackpy` in Python, and `TrackMate` in ImageJ/Fiji:

Detection

Each image was smoothed with a Gaussian kernel with a standard deviation (std. dev.) significantly less than the pixel-size of a feature (bright image formed by a single phage). A background image was generated by smoothing the image with a std. dev. significantly greater than the feature. The second image was subtracted from the first image, resulting in significantly sharper features. As most of the pixels did not contain a phage, the histogram of individual pixel intensities (calculated by pooling several images) was fit in the vicinity of its peak with a Gaussian distribution to extract the mean and std. dev. of the noise pixel intensities. All pixel intensities that were less than 2 noise std. dev. above the noise mean were set to zero. Most of the patches of non-zero pixels left in each frame contained phages. Patches consisting of a single non-zero pixel were set to zero intensity, because phages typically occupy several pixels. Patches in which the highest-intensity pixel was less than 5 noise standard deviations above the noise mean were also set to zero, only leaving pixel patches that contained phages in focus. Finally, the phage-positions were refined to sub-pixel resolution by fitting a 2D Gaussian profile to each phage image (16).

Tracking

As a first pass, starting from the first frame, for each detection in the frame, the closest detection in the next frame (frame gap $\Delta t = \frac{1}{30}\, s$) within a search radius of $r = \sqrt{4D_{max}\Delta t}$ was found, where D_{max} was set to 10 μm^2/s, which is significantly larger than 3 μm^2/s, the typical mean diffusion coefficient of a free phage particle (**Fig 1**). Then, for each detection in frame i, the closest detection in frame $i+1$ within search radius r was considered the same phage and linked into a trajectory. This process was repeated for each phage in the frame, and then for all frames, in

chronological order. Trajectories that lasted only a single frame were considered false positive detections and were removed.

The assignment procedure described above could leave gaps in the trajectory of a given phage if it disappeared from the field of view for a few frames, for example by diffusing out of the depth of field and immediately diffusing back in. Linking trajectories across these gaps allowed a more accurate estimation of Dwell time. To close such gaps, trajectories were looped over from longest to shortest for a given gap size of k frames. For each trajectory, trajectories that started k frames after (before) the current one within a distance $r(k) = k\sqrt{4D_{max}\Delta t}$ of the current trajectory's end (start) were searched. If multiple nearby trajectories were found, the closest one was linked to the current trajectory. The loop was then continued to the next trajectory. Trajectories for which links were made were then revisited. Once no more links were made at gap size k, the gap size was increased by 1 frame. This process was repeated up to a maximum of 3 frames (0.1 s). Finally, trajectories that lasted only two frames were considered false positives and were removed.

Multiplicity of infection (MOI) in microscopy assays

Multiplicity of infection (MOI) was calculated for each dataset (i.e., one snapshot of the immobilized bacterial cells + movie of fluorescently labeled phages interacting with those bacteria). Number of cells, N_b, was calculated from the phase-contrast image of bacteria. Number of phages, N_p was calculated as median number of detections in the first hundred frames of the fluorescent-phage movie. MOI was calculated as

$$MOI = \frac{N_p}{N_b}$$

<u>Classical adsorption assays</u>

Previously described standard protocol was followed (2). Briefly, cells were grown to OD_{600} ~0.5 and diluted to OD_{600} ~0.1-0.2. A known concentration of phages (~2-4×10^4 PFU/mL) was mixed with bacterial cells taken from an exponentially-growing culture at $t = 0$. The mixture was grown with 34°C incubation, the same temperature as in microscopy experiments, with gentle shaking (60 rpm). Aliquots were sampled from the culture every minute for ten minutes. Each aliquot was immediately added to chilled tubes (4 °C) containing 3-4 drops of 100% chloroform and the tube was thoroughly vortexed. 4 mL of molten 0.75% top agar and 100 μL overnight bacterial culture were added to the tube, vortexed, and poured on 1.5% bottom agar plate incubated at 37°C. Plaques visible after 16-24 hours were counted to estimate the number of free (non-attached) phage particle concentration at each time point. The effective adsorption rate constant k_{eff} was calculated by obtaining least square fits for

$$P = P_0 \exp\left(-k_{eff}\, t\right) \quad \text{Equation 1}$$

where P_0 is the initial concentration (PFU/mL) of phages, and t represents time that it takes for the phage concentration to decrease from P_0 to P (see **Supplementary Note 3** for derivation).

SI References

1. D. E. Deatherage, J. E. Barrick, "Identification of Mutations in Laboratory-Evolved Microbes from Next-Generation Sequencing Data Using breseq" in *Engineering and Analyzing Multicellular Systems: Methods and Protocols*, Methods in Molecular Biology., L. Sun, W. Shou, Eds. (Springer, 2014), pp. 165–188.
2. A. M. Kropinski, Measurement of the rate of attachment of bacteriophage to cells. *Methods Mol Biol* **501**, 151–155 (2009).
3. S. T. Abedon, Bacteriophage Adsorption: Likelihood of Virion Encounter with Bacteria and Other Factors Affecting Rates. *Antibiotics* **12**, 723 (2023).
4. Z. J. Storms, D. Sauvageau, Modeling tailed bacteriophage adsorption: Insight into mechanisms. *Virology* **485**, 355–362 (2015).
5. A. P. Krueger, The sorption of bacterio phage by living and dead susceptible bacteria. *J Gen Physiol* **14**, 493–516 (1931).
6. C. Kan, M. Le, "Physical and Theoretical Chemistry. Supplemental Modules: 2.8.1: Pseudo-1st-order Reactions" in (LibreTexts Online Textbooks), p. 2.8.1.
7. M. Delbrück, Adsorption of bacteriophage under various physiological conditions of the host. *J Gen Physiol* **23**, 631–642 (1940).
8. K. E. Kortright, R. E. Done, B. K. Chan, V. Souza, P. E. Turner, Selection for Phage Resistance Reduces Virulence of Shigella flexneri. *Applied and Environmental Microbiology* **88**, e01514-21 (2022).
9. B. K. Chan, *et al.*, Phage selection restores antibiotic sensitivity in MDR Pseudomonas aeruginosa. *Sci Rep* **6**, 26717 (2016).
10. L. L. Dreesens, *Illuminating the highly dynamic on-cell target search of bacteriophage and phage-like particles* (2021).
11. J. J. Dennehy, S. T. Abedon, "Adsorption: Phage Acquisition of Bacteria" in *Bacteriophages*, D. R. Harper, S. T. Abedon, B. H. Burrowes, M. L. McConville, Eds. (Springer International Publishing, 2021), pp. 93–117.
12. T. F. Anderson, The role of tryptophane in the adsorption of two bacterial viruses on their host, E. coli. *J Cell Compar Physiol* **25**, 17–26 (1945).
13. J. D. Antani, *et al.*, Mechanosensitive recruitment of stator units promotes binding of the response regulator CheY-P to the flagellar motor. *Nat Commun* **12**, 5442 (2021).
14. J. D. Antani, A. X. Sumali, T. P. Lele, P. P. Lele, Asymmetric random walks reveal that the chemotaxis network modulates flagellar rotational bias in Helicobacter pylori. *eLife* **10**, e63936 (2021).
15. H. H. Mattingly, K. Kamino, B. B. Machta, T. Emonet, Escherichia coli chemotaxis is information limited. *Nat. Phys.* **17**, 1426–1431 (2021).
16. R. Parthasarathy, Rapid, accurate particle tracking by calculation of radial symmetry centers. *Nat Methods* **9**, 724–726 (2012).
